# Sorption Behavior of Azo Dye Congo Red onto Activated Biochar from *Haematoxylum campechianum* Waste: Gradient Boosting Machine Learning-Assisted Bayesian Optimization for Improved Adsorption Process

**DOI:** 10.3390/ijms25094771

**Published:** 2024-04-27

**Authors:** Diego Melchor Polanco Gamboa, Mohamed Abatal, Eder Lima, Francisco Anguebes Franseschi, Claudia Aguilar Ucán, Rasikh Tariq, Miguel Angel Ramírez Elías, Joel Vargas

**Affiliations:** 1 Facultad de Ingeniería, Universidad Autónoma del Carmen, Ciudad del Carmen 24115, Campeche, Mexico; 100631@mail.unacar.mx; 2 Institute of Chemistry, Federal University of Rio Grande do Sul (UFRGS), Av. Bento Goncalves 9500, P.O. Box 15003, Porto Alegre 91501-970, RS, Brazil; eder.lima@ufrgs.br; 3 Facultad de Química, Universidad Autónoma del Carmen, Calle 56 No. 4 Av. Concordia, Ciudad del Carmen 24180, Campeche, Mexico; fanguebes@pampano.unacar.mx (F.A.F.); caguilar@pampano.unacar.mx (C.A.U.); mramirez.unacar@gmail.com (M.A.R.E.); 4 Tecnologico de Monterrey, Institute for the Future of Education, Ave. Eugenio Garza Sada 2501, Monterrey 64849, Nuevo León, Mexico; rasikhtariq@tec.mx; 5 Instituto de Investigaciones en Materiales, Unidad Morelia, Universidad Nacional Autónoma de México, Antigua Carretera a Pátzcuaro No. 8701, Col. Ex Hacienda de San José de la Huerta, Morelia 58190, Michoacán, Mexico; jvargas@iim.unam.mx

**Keywords:** activated biochar, azo dye, Congo red adsorption, *Haematoxylum campechianum*, isotherms, educational innovation, computational thinking

## Abstract

This work aimed to describe the adsorption behavior of Congo red (CR) onto activated biochar material prepared from *Haematoxylum campechianum* waste (*ABHC*). The carbon precursor was soaked with phosphoric acid, followed by pyrolysis to convert the precursor into activated biochar. The surface morphology of the adsorbent (before and after dye adsorption) was characterized by scanning electron microscopy (SEM/EDS), BET method, X-ray powder diffraction (XRD), and Fourier-transform infrared spectroscopy (FTIR) and, lastly, pH_pzc_ was also determined. Batch studies were carried out in the following intervals of pH = 4–10, temperature = 300.15–330.15 K, the dose of adsorbent = 1–10 g/L, and isotherms evaluated the adsorption process to determine the maximum adsorption capacity (Q_max_, mg/g). Kinetic studies were performed starting from two different initial concentrations (25 and 50 mg/L) and at a maximum contact time of 48 h. The reusability potential of activated biochar was evaluated by adsorption–desorption cycles. The maximum adsorption capacity obtained with the Langmuir adsorption isotherm model was 114.8 mg/g at 300.15 K, pH = 5.4, and a dose of activated biochar of 1.0 g/L. This study also highlights the application of advanced machine learning techniques to optimize a chemical removal process. Leveraging a comprehensive dataset, a Gradient Boosting regression model was developed and fine-tuned using Bayesian optimization within a Python programming environment. The optimization algorithm efficiently navigated the input space to maximize the removal percentage, resulting in a predicted efficiency of approximately 90.47% under optimal conditions. These findings offer promising insights for enhancing efficiency in similar removal processes, showcasing the potential of machine learning in process optimization and environmental remediation.

## 1. Introduction

Water pollution is a serious concern because almost all industrial activities generate pollutants that are discharged into water resources without treatment or with a deficiency treatment. Dyes are an example of stable organic compounds used in many industries, such as textile, paper, plastic, printing, cosmetics, and others. When dyes reach aquatic environments, they risk the aquatic ecosystem and even human health because some are toxic, carcinogenic, and mutagenic [1]. In addition, small amounts of dye cause a change in the aesthetic perception of the water and also affect the penetration of sunlight and the amount of oxygen dissolved in water [2].

Many types of dyes are on the market, and their classification depends mainly on how they are applied to the substrate and their chemical structure. The first one includes direct dyes, reactive dyes, vat dyes, and disperse dyes, and the latter includes azo dyes, indigo dyes, acid dyes, basic dyes, and anthraquinone dyes, among others [3]. About 50% of the dyes on the market are azo dyes, which is why azo dyes are considered the most important widely used dyes [4].

Congo red (CR) is a di-azo dye containing two azo groups (-N=N-) which are attached to two aromatic radicals. CR is widely used in the textile industry due to the fact that it can easily form complexes with polysaccharides such as cellulose [5,6]. Another application of Congo red is in histology, identifying amyloid proteins in tissues to diagnose amyloidosis [7].

In the aqueous solution, the molecular structure of CR is affected by pH variations, which give different colors. Congo red solutions remain red at neutral and alkaline pH values but turn blue at acid pH values [6]. Thus, CR is also used as an acid–base indicator in several industries. As mentioned above, CR dye has different uses in various industries that generate waste that can be discharged into water resources. The problem with azo dyes is their toxicity; when they degrade under anaerobic conditions, the benzidine formed is a known carcinogen. Therefore, benzidine is a byproduct of CR degradation that may cause bladder cancer in humans [8]. In addition to their carcinogenic effect, azo dyes have other toxic effects that can affect human health by damaging organs such as skin, kidneys, liver, and even central nervous system [9].

According to its adverse effects on humans and plants [10,11], Congo red dye presents a significant risk to human health and the environment, so it is vital to achieve its elimination from aqueous media. There are many technologies for removing dyes, particularly for Congo red dye in aqueous solution. The most common methods applied are biological degradation, photocatalysis, and adsorption. Microorganisms have developed resistance to high dye concentrations and may degrade them into carbon dioxide, inorganic salts, and water by enzymatic action. Different species of genus *Aspergillus* fungi, such as *Aspergillus flavus* [12], *Aspergillus niger* [13], and *Aspergillus fumigatus* [14], have been successfully used to biodegrade CR. Photocatalysis is an advanced removal technology that has received more attention recently due to its main advantages, such as rapid dye degradation and no generation of secondary waste [15]. Recent and novel investigations to synthesize new photocatalysts and apply them to Congo red removal have been carried out, such as orange peel extract biosynthesized zinc oxide nanoparticles being used to remove CR via photocatalysis [16] and novel rare earth metal doped ZnO photocatalysts for degradation of CR [17].

Adsorption has also been used for the removal of CR. Some biosorbents such as *Antigonon leptopus* leaf powder [18], mango leaves powder [19], *Moringa oleifera* seed coat [20], and peel waste of *Hylocereus undatushave* (white dragon fruit) [21] have been used to remove CR dye efficiently. On the other hand, the most used adsorbent is commercial activated carbon due to its high uptake capacity, high surface area, and environmental friendliness. Non-toxicity is one of its advantages. However, its main disadvantage is its high operating costs [22]. So, a need for new materials for synthesizing activated biochars has risen. Different biomass, such as Guar gum [23] and walnut shell [24], have been used to produce activated carbon.

In this work, activated biochar was prepared from *Haematoxylum campechianum* waste, taking advantage of its abundance in the state of Campeche in Mexico. This material was used to remove the azo dye CR in aqueous solution. *ABHC* was characterized before and after adsorption of the dye by SEM/EDS, BET method, X-ray powder diffraction (XRD), and Fourier-transform infrared spectroscopy (FTIR), and, lastly, pH_pzc_ was also determined. The effects of contact time, dye concentration, temperature, pH of the aqueous medium, and the activated biochar dose were evaluated to compare and evaluate the removal efficiency. Cycles of adsorption–desorption were performed to determine the reusability of the activated biochar.

Another research contribution of this work is to present a novel approach to optimize chemical removal processes using advanced machine learning techniques. The efficient removal of contaminants from various mediums is a critical challenge in environmental engineering and remediation. Traditional optimization methods often rely on labor-intensive experimentation and trial-and-error approaches. Here, we contribute to the field by proposing a data-driven approach that leverages the power of Gradient Boosting regression models and Bayesian optimization. By harnessing a rich dataset containing information on initial concentration, time, temperature, pH, and dose, we aim to maximize the removal percentage through precise tuning of input variables. This research not only offers a pragmatic solution to enhance removal efficiency but also underscores the potential of machine learning in addressing complex environmental challenges.

## 2. Results and Discussion

### 2.1. Characterization of ABHC

**Figure 1:**
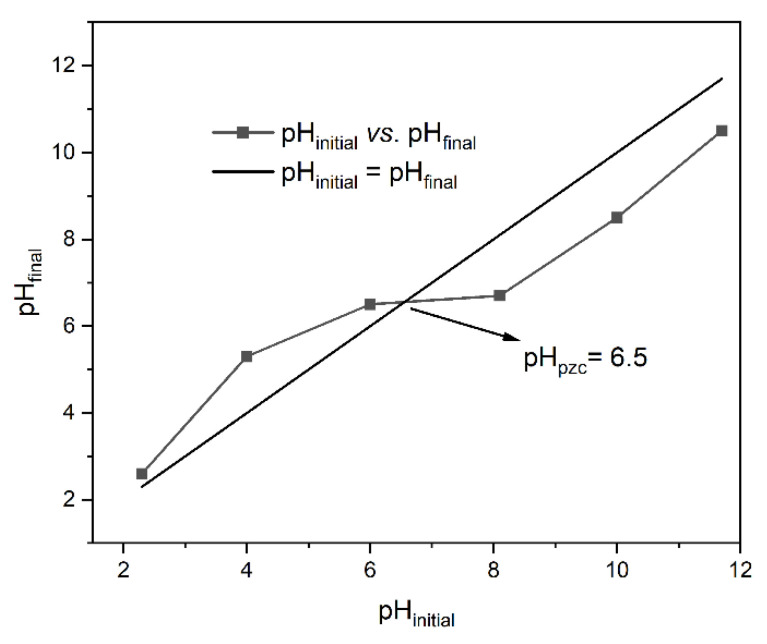
pH_pzc_ of *ABHC*.

As shown in Figure 1, the point of zero charge of *ABHC* was 6.5. This result is similar to those obtained from other biomass precursor materials such as *Dipterocarpus alatus* (pH_pzc_ = 6.3) [25] and rice husk (pH_pzc_ = 6.8) [26]. Therefore, when the solution pH is above the pH_pzc_ (pH > pH_pzc_), the surface of *ABHC* is negatively charged and the cationic species will be preferentially removed. In contrast, if values of pH are below pH_pzc_ (pH < pH_pzc_), the charge of the surface of *ABHC* will become positive, and then anionic species are preferentially attracted via electrostatic interactions [27].

**Figure 2:**
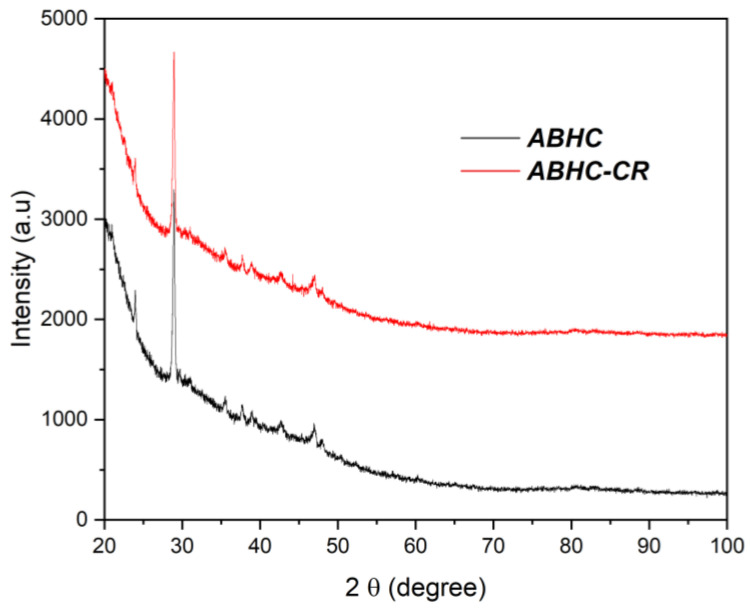
XRD pattern of *ABHC* and *ABHC*-CR.

XRD patterns of *ABHC* before and after Congo red adsorption are presented in Figure 2. For *ABHC*, there is a highest intensity diffraction peak around 2*θ* = 26.5, which can be assigned to the crystalline hexagonal phase of graphite [28]. There are other diffraction peaks, with minor intensity, around 2*θ* = 22.3 and 42.9, which are attributed to crystalline graphite [29]. After CR adsorption, the diffraction pattern did not present significant changes, indicating that there is no change in the crystallinity of *ABHC* as a result of the adsorption process [24]. Similar results were observed in previous studies. Homagai et al. (2022) reported that the XRD patterns did not show remarkable changes after adsorption of crystal violet on modified rice husk. This result was due to the amorphous nature attributed to the lignin, cellulose, and hemicellulose present in the biosorbents [30]. Furthermore, Li et al. (2020) reported that there was no change in XRD patterns after adsorption of Congo red and methylene blue dye on walnut shell-based activated carbon and also attributed their result to the amorphous nature of the adsorbent [24]. Therefore, it can be assumed that the similarity on XRD diffraction patterns of *ABHC* before and after Congo red adsorption could be attributed to the presence of lignin, cellulose, and hemicellulose, which are confirmed by FT-IR.

The grain size of the *ABHC* and *ABHC-CR* samples was calculated from the XRD patterns using the Debye–Scherrer Equation (1).

(1) $$D=\frac{K\lambda }{BCos(\theta_{B})}$$

where *λ* is the wavelength (λ(CuK_α_) = 0.15405 nm), *K* is an empirical constant related to the crystalline shape (for no spherical shapes, *K* = 0.9), *B* is the full width at half maximum of the peak (FWHM) in radian on the 2*θ* scale, and *θ* is the Bragg’s diffracting angle corresponding to the maximum of the peak. FWHM were determined using the Origin Pro version 2021 based on the Gaussian line.

According to Equation (1), the average particle size of the *ABHC* and *ABHC*-CR samples was 25.56 nm and 25.69 nm, respectively. This result implies that the particle size of AC was not affected after adsorption of CR.

Scanning electron microscope (SEM) analysis was carried out to investigate the physical surface morphology of *ABHC* before and after dye adsorption.

**Figure 3:**
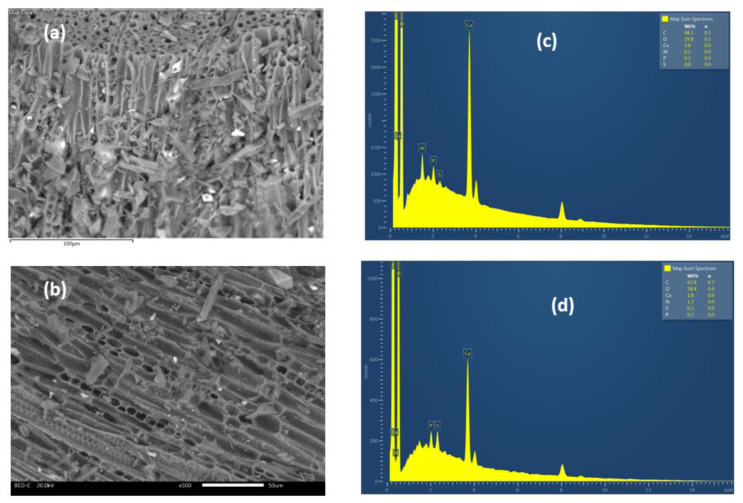
SEM images of (**a**) *ABHC* and (**b**) *ABHC*-CR. EDS results of (**c**) *ABHC* and (**d**) *ABHC*-CR.

The SEM micrographs of *ABHC* (Figure 3a) show that the particles of the synthesized biochar material before dye adsorption have a rough surface and an irregular shape with a variety of randomly distributed cavities, which can provide easy access transport toward the adsorption sites [31]. After Congo red adsorption, *ABHC* has slight changes on its surface, and some cavities were filled with CR molecules. Additionally, SEM micrographs after CR adsorption (Figure 3b) show new flake-like deposits formed on the activated biochar surface due to the interactions between the functional groups on the activated biochar and the dye molecule. The elemental composition of the biochar material was performed by energy dispersive X-ray spectroscopy (EDS), as shown in Figure 3c. In *ABHC*, the material consists predominantly of carbon and oxygen, and the sum of these two elements is 98.0% per weight. The rest of the composition (2.0%) corresponds to metallic fractions (Ca, Al, and P). Lastly, the elemental analysis presents 1.7% per weight of N and 0.2% of S (Figure 3d). Nitrogen is found in the Congo red molecule (azo dye). Additionally, sulfur is also found in the molecule of the dye (Figure 16), which confirms the adsorption of dye onto the adsorbent.

**Figure 4:**
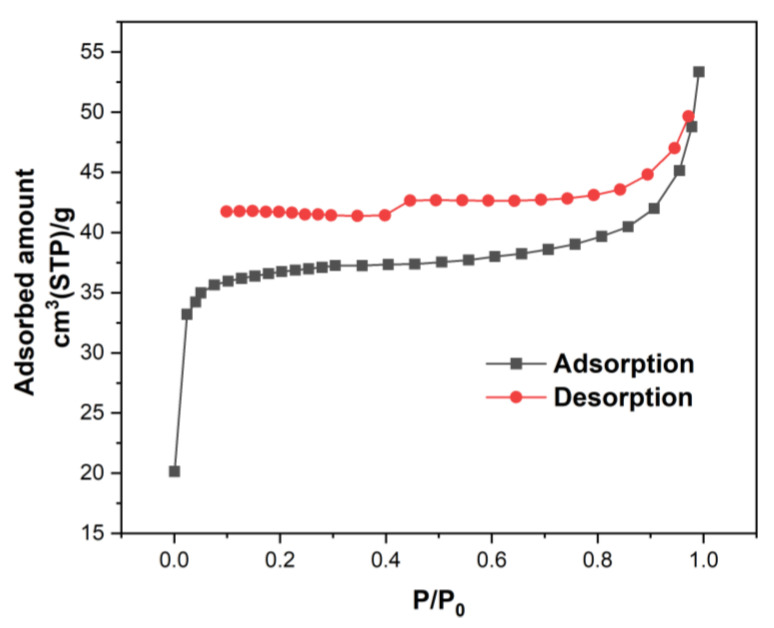
Adsorption–desorption isotherm of N_2_ on the *ABHC*.

Figure 4 shows the adsorption–desorption isotherm of N_2_ on the *ABHC* sample. As observed in this figure, the isotherm belonged to type IV according to International Union of Pure and Applied Chemistry (IUPAC classification), indicating that the *ABHC* had mesoporous structure [32].

The mean pore diameter of *ABHC* calculated by Barrett, Joyner, and Halenda (BJH) equations was 2.14 nm, with a surface area of 124.15 m^2^/g. The difference in the surface properties can be attributed to the type of biomass precursor. The mean pore diameter value of biochar allows it to be classified as a mesoporous type, and several activated carbons with mesoporous type have been used to remove Congo red dye effectively [33]. Additionally, the width of the CR molecule is approximately 2.62 nm [34], requiring a mesoporous type for being adsorbed. Lafi et al. (2019) [35] prepared activated carbon using coffee waste as a precursor and reported a mean pore diameter of 4.04 nm and surface area of 219.69 m^2^/g and removed CR efficiently. Values of pore diameter and surface area of *ABHC* are close to those mentioned above. Therefore, it is suspected that *ABHC* can be used to remove CR dye from the aqueous solution.

**Figure 5:**
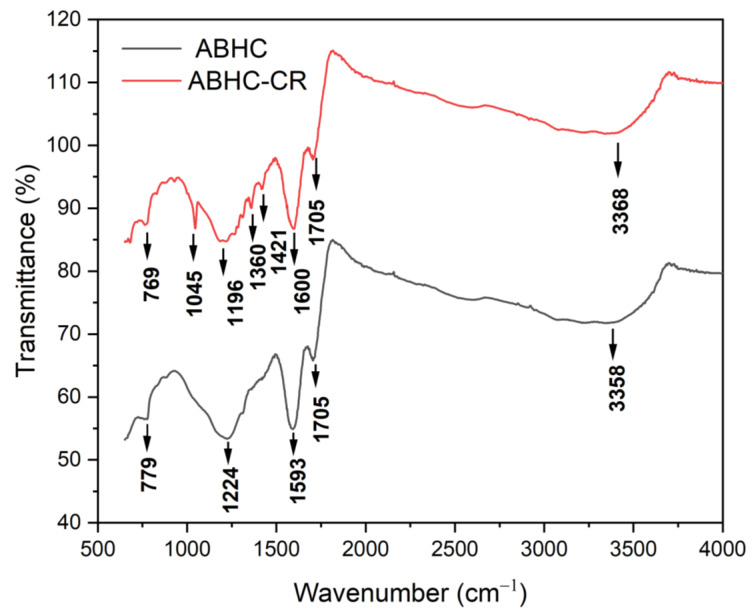
FT-IR of *ABHC* before and after CR adsorption.

Figure 5 shows the FTIR spectrum for *ABHC* and *ABHC*-CR, and the assigned bands are summarized in Table 1. For activated biochar from *Haematoxylum campechianum* (*ABHC*) broadband at 3358 cm^−1^ attributed to OH groups present in cellulose [33], the band at 1705 cm^−1^ could be attributed to C=O present in lignin [5], and the band at 1224 cm^−1^ can represent C-O stretching of aryl, carboxylates, or ether groups presents in lignin [33]. The groups mentioned above are known to participate in pollutant adsorption processes. After Congo red adsorption, new bands appeared. At 1421 cm^−1^, this band can be attributed to C=C stretching in the aromatic ring [36], and the band at 1045 cm^−1^ corresponds to S=O [37]. These functional groups are present in the Congo red dye molecule (Figure 16) and confirm the presence of the adsorbate in the adsorbent. The change in the bands from 3358, 779, and 1224 cm^−1^ to 3368, 769, and 1196 cm^−1^ indicates that OH, N-H, and C-O groups are mainly involved in the adsorption of CR onto *ABHC* and suggests hydrogen bonding, n–π, and π–π interactions [38] as part of the dye adsorption interactions.

### 2.2. Kinetic Study

**Figure 6:**
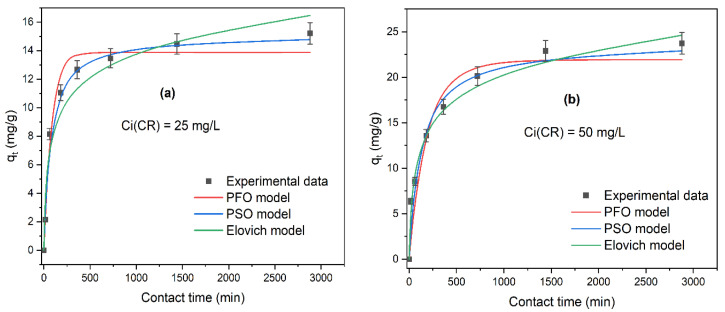
Kinetic modeling of Congo red adsorption (**a**) C_i_ = 25 mg/L and (**b**) C_i_ = 50 mg/L pseudo-first-order, pseudo-second-order, and Elovich nonlinear models by *ABHC* (pH = 7; D *ABHC* = 1 g/L; T = 300.15 K; contact time = 15–2880 min).

The behavior of the experimental data and their fit to the kinetics models are shown in Figure 6. For both initial concentrations (25 and 50 mg/L), equilibrium seems to be reached slowly, around 24 h of contact, and the experimental data are closer to the pseudo-second-order model (blue curve) (Figure 6a,b). The results of the fit to the kinetic models and the corresponding parameter values are shown in Table 2.

For C_i_CR = 25 mg/L, the three models presented a good fit to the experimental data with values of R^2^ > 0.900. PFO, PSO, and Elovich models show values of R^2^ = 0.957, R^2^ = 0.988, and R^2^ = 0.928, respectively. However, error functions show better results for PSO (Table 3). Additionally, the adsorption capacity at equilibrium (q_e,calc_) obtained with the pseudo-second-order model was 15.09 mg/g and is closer to the experimental adsorption capacity at equilibrium (q_e,exp_ = 15.21 mg/g) than q_e_ obtained with the pseudo-first-order model (13.87 mg/g). Therefore, the best model to describe the kinetics of the adsorption of Congo red at C_i_CR = 25 mg/L is the pseudo-second-order.

Figure 6b shows a good fit to the PSO and Elovich adsorption kinetic models at an initial concentration (C_i_CR = 50 mg/L) with values for R^2^ > 0.900 and small values for the error functions (Table 3). The adsorption capacity at equilibrium (q_e,cal_) obtained with the pseudo-second-order (PSO) model was 23.81 mg/g and is closer to q_e,exp_ = 23.74 mg/g than q_e,cal_ obtained with the pseudo-first-order PFO model. Furthermore, it is important to mention the good fit with the Elovich model (R^2^ = 0.968), which assumes that the activation energy increases with adsorption time and that the surface of the adsorbent is heterogeneous [39]. In summary, the adsorption kinetics of CR dye at the two initial concentrations (25 and 50 mg/L) follow pseudo-second-order (PSO). A tendency to fit the pseudo-second-order model at low initial dye concentrations is observed. Sabarinathan et al. (2019) [40] reported the same trend in the adsorption kinetics of methylene blue onto molecular polyoxometalate, and Ho & Mckay (1998) [41] studied the adsorption of two dyes, Basic Blue 69 and Acid Blue 25, onto peat. According to the results of the kinetic study, the capacity of adsorption at equilibrium (q_e,exp_) does not increase considerably between 24 and 48 h. Hence, the next equilibrium experiments were carried out at the equilibrium time of 24 h.

### 2.3. Adsorption Isotherms of CR at Different Solution pH

**Figure 7:**
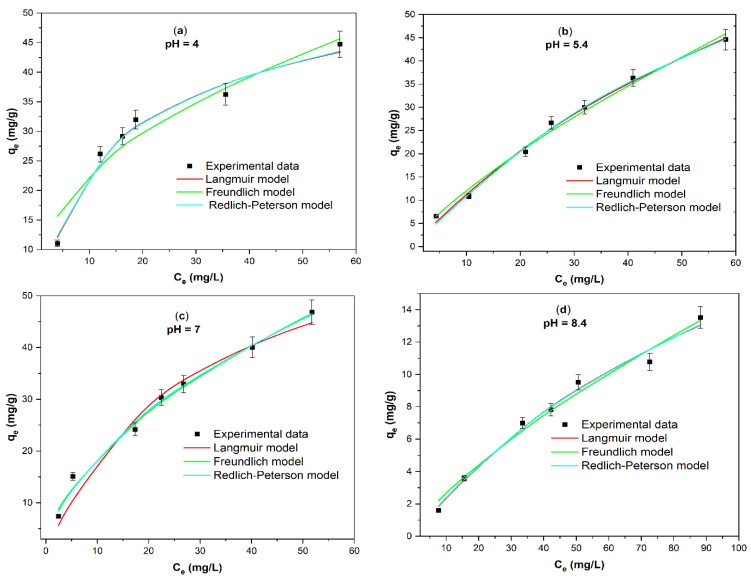

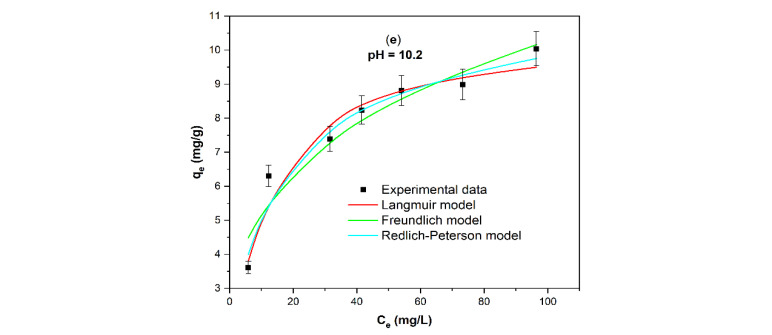
Adsorption isotherms modeling of Congo red at different pH values: (**a**) pH = 4.0, (**b**) pH = 5.4, (**c**) pH = 7.0, (**d**) pH = 8.4, and (**e**) pH = 10.2 (T = 300.15 K; C_i_CR = 10 mg/L–100 mg/L; dose = 1 g/L; contact time = 1440 min).

Congo red is used as a pH indicator. At acidic pH values (2.18–3.16), CR solutions turn blue, and there is a shift in the maximum absorbance wavelength [42], and at pH ≥ 3.86, there is no significant shift in the maximum absorbance wavelength. Hence, the study of the effect of pH was carried out in the range of 4 to 10 (Figure 7). According to the best values of the determination coefficient R^2^ (Table 4) and error functions (Table 5), the Redlich–Peterson and Langmuir models fit best over the entire studied pH range. β values from the Redlich–Peterson model are close to 1. Therefore, the fit to the Langmuir model is further supported, and it can be assumed that the adsorption of Congo red onto activated biochar prepared from *Haematoxylum campechianum* waste (*ABHC*) implies the following considerations: all the adsorption sites are equal; each adsorption site only keeps one molecule of the adsorbate and all sites do not sterically and energetically depend on the adsorbed amount of the adsorbate [43]. Table 4 shows the maximum adsorption capacity, Q_max_ = 53.93, 114.8, 68.33, 30.83, and 10.51 mg/g at pH = 4.0, 5.4, 7.0,8.4, and 10.2, respectively. At high pH values (7.0, 8.4, and 10.2), Q_max_ values decrease. This behavior can be attributed to the pK_a_ value of Congo red = 4.5 [44], which means that CR would be negatively charged at basic values of pH solution and also at the pH_pzc_ of *ABHC*, where, at pH > 6.5, the surface of *ABHC* is negatively charged. Therefore, there will be repulsion between the adsorbent surface’s negative charge and the adsorbate’s negative charge. A decrease in Q_max_, from 114.8 to 53.93 mg/g at pH from 4.0 to 5.4 was also observed. This decrease is due to the positive charge of *ABHC* (pH < pH_pzc_ = 6.5) and the protonation of CR, resulting in repulsion. The highest value of Q_max_= 114.831 mg/g was obtained at a pH solution of 5.4, suggesting the best adsorption of CR at this pH value. Other works have reported an optimal pH value for the removal of CR around 5 [27,45,46].

### 2.4. Adsorption Isotherms of CR at Different Adsorbent Dose

**Figure 8:**
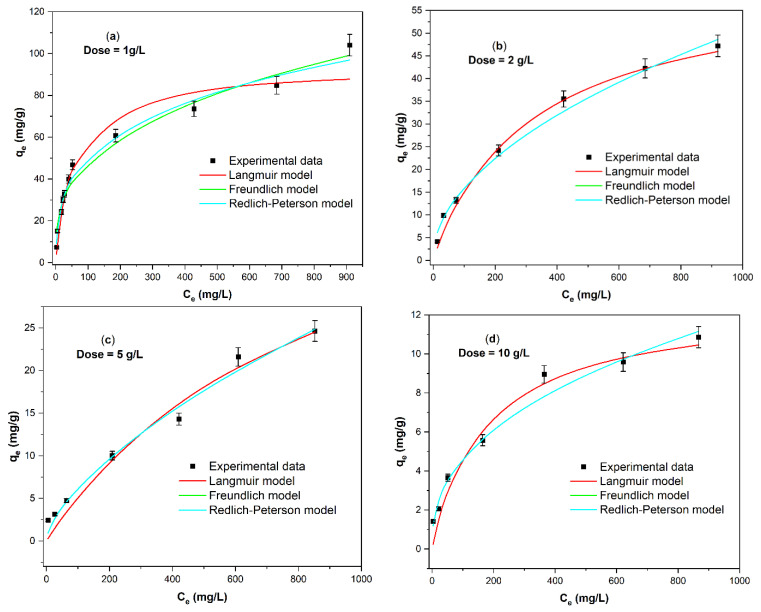
Adsorption isotherms modeling of Congo red at different *ABHC* doses: (**a**) dose = 1 g/L, (**b**) dose = 2 g/L, (**c**) dose = 5 g/L, and (**d**) dose = 10 g/L (pH = 7; C_i_CR = 20–1000 mg/L; T = 300.15 K; contact time = 1440 min).

Figure 8 depicts the effect of the dose of *ABHC* on the adsorption of Congo red dye. It is observed that for all doses (1 g/L to 10 g/L), the experimental data are close to Freundlich, Langmuir, and Redlich–Peterson nonlinear isotherms; however, for low doses (1 g/L and 2 g/L), the experimental data are also close to mode. In addition, a decrease in the adsorption capacity at equilibrium (q_e_) is observed when the dose of activated biochar increases (Figure 8a–d).

The parameters of each adjusted nonlinear isotherm model at a different dose of *ABHC* are listed in Table 6. At low dose values (1 g/L and 2 g/L), the values of R^2^ were superior to 0.900 for all isotherm models. For Langmuir isotherm, the R^2^ values were 0.940 and 0.988, while for Freundlich, R^2^ = 0.976 and 0.993 for doses of 1 g/L and 2 g/L, respectively. The good fit to both models suggests that the adsorption of CR dye onto activated biochar involves several interactions, which had already been mentioned previously in FTIR studies. At high doses of *ABHC* (5 g/L and 10 g/L), the best adjustments are obtained with the Langmuir (R^2^ = 0.970 and R^2^ = 0.966), Freundlich (R^2^ = 0.985 and R^2^ = 0.982), and Redlich–Peterson (R^2^ = 0.985 and R^2^ = 0.982).

In the whole range of doses (1 g/L to 10 g/L), the best-fitted model was Redlich–Peterson with the highest values of R^2^ and the lowest values of error functions (Table 7); this is a hybrid model of the Langmuir and Freundlich models, and when its dimensionless parameter β is close to one, the model approaches Langmuir, and when β is close to zero, the model approaches Freundlich [47]. At low dose, dose = 1 g/L, β = 0.742 and at dose = 2 g/L, β = 0.669 values are close to 1, so adsorption is close to the features of the Langmuir model. By increasing the dose to 5 g/L (β = 0.367 is closer to zero), adsorption is close to the features of the Freundlich model. Finally, Table 6 shows that Q_max_ (from the Langmuir isotherm model) decreases as the dose of activated biochar increases (from Q_max_ = 92.86 mg/g at dose = 1 g/L to Q_max_ = 12.22 mg/g at dose = 10 g/L); this behavior is due to the fact that as the dose of *ABHC* increases, there is a decrease in the amount of free active sites because of the agglomeration of sorption sites at high dose [9] or the solute concentration gradient between the solution of dye and the adsorbent surface, so the amount of CR adsorbed per unit weight of *ABHC* is reduced with increasing *ABHC* dose [48]. The highest value of the parameters Q_max_= 92.86 mg/g and n = 3.079 were achieved with the lowest dose used in this work; therefore, the optimum dose of *ABHC* is 1 g/L.

### 2.5. Adsorption Isotherms of CR at Different Temperatures

**Figure 9:**
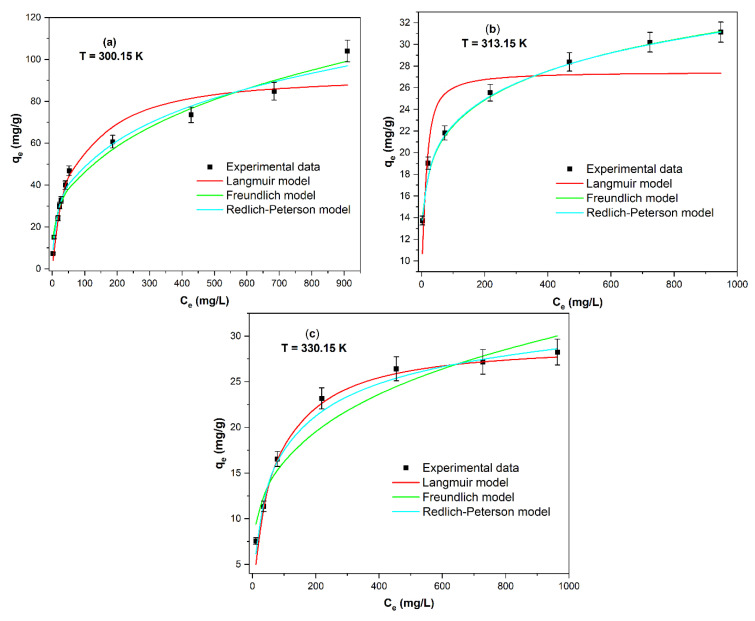
Adsorption isotherms modeling of Congo red at different temperatures: (**a**) T = 300.15, (**b**) T = 313.15, (**c**) T = 330.15 (pH = 7; C_i_CR = 20–1000 mg/L; dose = 1 g/L; contact time = 360 min).

The effect of the temperature on the adsorption of Congo red is shown in Figure 9. A change in the shape of the isotherm is observed when temperature increases from T = 300.15 to T = 330.15. In the whole studied temperature range, experimental data best fit the Redlich–Peterson model.

At low temperatures (Figure 9a), experimental data are closer to the Freundlich nonlinear model than the Langmuir nonlinear model. However, at higher temperatures (Figure 9c), experimental data and the shape of the isotherm are closer to the nonlinear Langmuir model than the Freundlich nonlinear model.

Table 8 summarizes the parameters of the Langmuir, Freundlich, and Redlich–Peterson nonlinear isotherms. The maximum adsorption capacity, Q_max_ (mg/g) (from the Langmuir model), decreased significantly from 92.86 to 29.20 mg/g at 300.15 and 330.15 K, respectively. In other words, the adsorption of CR onto *ABHC* is less favorable at high temperatures. In addition, K_F_ (from the Freundlich model) values decreased when increasing the temperature from 300.15 to 330.15 K, which also indicates unfavorable adsorption at high temperatures. This behavior has been commonly reported in the adsorption of azo dyes, suggesting a better adsorption of these types of dyes at low temperatures [49].

According to R^2^ and error function values (Table 9), at 300.15 and 313.15 K, the best fit to the isotherm models corresponds to the following order: Redlich–Peterson > Freundlich >Langmuir. While, at 330.15 K, Redlich–Peterson > Langmuir> Freundlich. That is, the best isotherm model to describe the adsorption of Congo red dye onto *ABHC* in the full range of studied temperatures is the Redlich–Peterson, which assumes the considerations of Freundlich and Langmuir models [47]. However, values of the parameter β presented an increasing trend as the temperature increased (β = 0.742 at T = 300.15 K and β = 0.899 at T = 330.15 K). Therefore, the dye adsorption tends to be in the Langmuir model at high temperatures.

### 2.6. Comparison of the Maximum Adsorption Capacity Q_max_ onto Different Activated Carbons

As mentioned in the introduction, CR dye is difficult to remove from the aqueous medium; this proves the low maximum adsorption capacity obtained with other activated carbons (Table 10). However, [50,51] and this work have reported high values of Q_max_ (Q_max_ > 100 mg/g), which indicates these activated carbons are good sorbents to remove CR. In Table 10, the experimental conditions are also shown. It is observed that most of the works reported Q_max_ at temperatures that are not too high, in the range of 25–30 °C, like this work. With respect to the pH value, high pH values are not observed, i.e., high adsorption capacities are not obtained at very high pH (as mentioned previously in the pH effect). However, Q_max_ values reported at lower pH values (2–3) are sometimes questioned due to the change in the wavelength of maximum absorbance of Congo red at acidic pH values that can lead to errors at the moment of quantifying CR solutions [42]. Activated biochar from *Haematoxylum campechianum* (*ABHC*) presented a high adsorption capacity of CR dye at not-so-extreme experimental conditions compared to the other activated carbons.

### 2.7. Adsorption and Desorption Cycles of CR

**Figure 10:**
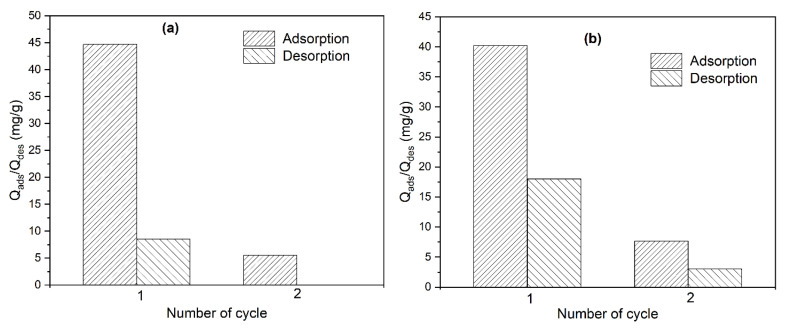
Effect of different effluent concentrations on biosorption/desorption cycles of CR onto *ABHC* (**a**) 0.01M NaOH and (**b**) 0.2M NaOH.

Two different NaOH concentrations (0.01 and 0.2 M) were used in two adsorption/desorption cycles. For 0.01M NaOH (Figure 10a), Q_ads_ decreased from 44.70 mg/g to 5.50 mg/g for cycles 1 and 2, respectively. Regarding the Q_des_, after cycle number 1, only 8.58 mg/g of CR was desorbed, and after cycle number 2, no desorption of CR was achieved. While, for 0.2M NaOH (Figure 10b), the adsorption capacity (Q_ads_) resulted in 7.69 mg/g for cycle number 2; comparing these results with those mentioned above for 0.01M NaOH, there is only an increase of approximately 2 mg/g in the adsorption capacity using 0.2M NaOH. Whereas, for the desorption capacity, after cycle number 1, Q_des_ increased from 8.58 mg/g to 18.01 mg/g, and for cycle 2, a desorption capacity of 3.02 mg/g was achieved, suggesting better desorption of Congo red with increasing NaOH concentration. However, these desorption results are low, which means that CR is well sorbed onto the surface of the *ABHC* and that the interactions are strong. The low adsorption removal after two cycles indicates the low possibility of reuse, which is a disadvantage of activated carbon that has already been reported [57]. In order to improve the reusability of *ABHC,* it is suggested to apply any other regeneration method such as another eluent or even temperature.

### 2.8. Machine Learning-Assisted Optimization for Improving the Adsorption Process

Machine learning (ML) techniques have become pivotal in enhancing the predictability [58] and understanding of adsorption processes across various domains, including environmental and materials science. This review synthesizes recent advancements in the integration of ML with adsorption modeling, highlighting its application in predicting the behavior of diverse adsorbents and adsorbates, from heavy metals to organic pollutants and gases. Guanwei Yin et al. (2021) explored different ML models—Multi-Layer Perceptron (MLP), Passive Aggressive Regression, and Decision Tree Regressor—to predict dye adsorption from aqueous solutions. Among these, the Decision Tree Regressor emerged as the most effective, suggesting its suitability for correlating adsorption equilibrium data due to high accuracy and low error rates (R^2^ = 0.99) [59]. Chen Zhao et al. (2024) demonstrated how multivalent ions like Ca^2+^, K^+^, Na^+^, and Mg^2+^ impact the adsorption of azo dyes using ML models coupled with Density Functional Theory (DFT). Their study revealed that the Gradient Boosting Decision Tree model provided the best fit, suggesting that mixed background ions significantly influence adsorption behavior, as validated through experimental and theoretical analyses [60]. Shoushi Zhao et al. (2024) applied ML to predict adsorption of various metal cations by clay minerals, identifying key descriptors through extensive feature engineering. The Extreme Gradient Boosting model was highlighted for its exceptional predictive accuracy (R^2^ = 0.977), underscoring the model’s capability in geochemical applications [61]. Qing-Yun Cai et al. (2024) focused on predicting protein adsorption capacities using QSAR models derived from ML. Utilizing Random Forest and Gradient Boosting methods, they achieved high predictive performances across various datasets, showing that ML can significantly contribute to the design of more efficient separation processes in biochemical engineering [62]. Hyeonmin Lee and Yongju Choi (2023) developed ML models to predict the adsorption capacity of sediment-amended activated carbon for hydrophobic organic contaminants. The top-performing model achieved an R^2^ of 0.94, illustrating the utility of ML in enhancing the predictability of adsorption processes in complex environmental matrices [63]. Lisheng Guo et al. (2024) employed multiple ML algorithms to model heavy metal adsorption by bentonite, with the extreme Gradient Boosting Regression (XGB) model showing the best performance. This study also provided insights into the influence of various factors on adsorption capacity, supported by a web-based graphical interface for model access [64]. Kai Chen et al. (2023) developed ML models to predict the adsorption percentage of oxyanions on goethite, assessing the influence of specific surface area and other descriptors. The study provided detailed insights into the non-linear relationships and crystal face compositions affecting adsorption capacities [65]. Raja Selvaraj et al. (2024) utilized ANN and ANFIS to model tetracycline adsorption on activated carbon derived from fruit biomass. This approach not only yielded high correlation coefficients but also provided a detailed mechanism of adsorption through statistical physics models, highlighting the method’s applicability in pharmaceutical waste treatment [66]. The reviewed studies collectively underscore the transformative potential of machine learning in adsorption science. By effectively predicting adsorption capacities and understanding the underlying mechanisms through models like Decision Trees, Gradient Boosting, and Extreme Gradient Boosting, ML facilitates more precise control and optimization of adsorption processes across varied applications. This integration not only enhances model accuracy but also contributes significantly to environmental sustainability and material efficiency.

**Figure 11:**
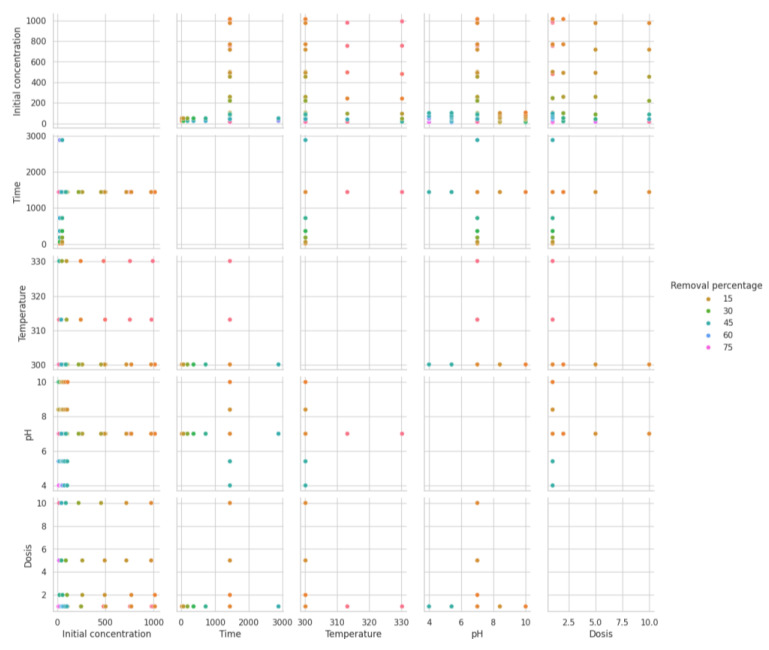
Scatter matrix showing the relationship between the input variables and the removal percentage.

The scatter matrix provided in Figure 11 presents a visual representation of pairwise relationships across multiple variables, with dot colors corresponding to different ranges of the “Removal percentage”. From the distribution of points, it can discern patterns and correlations within the data. The descriptive statistics complement the scatter matrix by quantifying the central tendency and dispersion of the same variables. With a count of 105 for each variable, it is a sizeable dataset. The “Initial concentration” has a mean of approximately 201.55, but its standard deviation is relatively high, at 293.31, indicating a wide range of values, which is also visible in the scatter plot with a spread across the axis. The “Time” variable shows less variation relative to its mean, suggesting more consistent data. The “Temperature” data are less varied, with a small standard deviation around its mean of 303.02. The “pH” values have a mean near neutral, at 7.01, and “Dosis” averages around 1.93 with a larger standard deviation, showing some variability in the dataset. The “Removal percentage” has a mean of 35.71, with a broad standard deviation of 22.47, which indicates variability in the removal efficiency. In the scatter matrix, there does not seem to be a clear linear pattern or strong correlation between most variables and the “Removal percentage”, as indicated by the wide scatter of points. However, a higher concentration of points along the lower range of “Initial concentration” suggests that lower concentrations may be associated with a broader range of “Removal percentage” outcomes. “Time” seems to show clusters at specific intervals, perhaps indicative of experimental time points or specific durations at which data were collected. Temperature appears fairly constant except for a few higher measurements, which might represent a different experimental condition. The pH levels are centered around neutral, with fewer instances of more acidic or basic conditions. The dosages show a strong clustering at the lower end, suggesting most experiments used a smaller dose, with fewer instances using higher dosages. Overall, the combination of scatter matrix and descriptive statistics provides a comprehensive overview of the dataset, indicating a wide variance in some variables and suggesting potential trends that might warrant further investigation, such as the influence of “Initial concentration” on the “Removal percentage”. These insights could be crucial for optimizing conditions to enhance the removal process’s efficiency.

**Figure 12:**
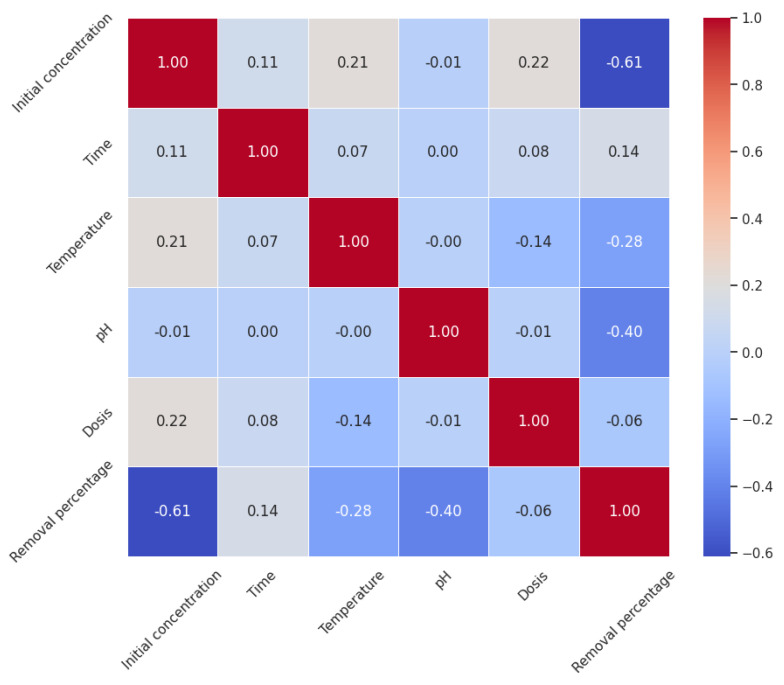
Heatmap based upon coefficient of correlation.

The heatmap visualization of this matrix, as constructed with seaborn and matplotlib, implementing Pearson Coefficient of Correlation, enhance the comprehensibility of these relationships, presenting a clear and concise graphical representation of the data, where the ‘coolwarm’ color palette effectively differentiates between positive and negative associations, displayed in Figure 12. In examining the correlation matrix for the dataset, there is a notable linear relationship between the variables and the removal percentage, which serves as the target variable in the context of the removal process’s efficacy. The initial concentration is significantly inversely correlated with the removal percentage (r = −0.61), indicating a tendency for the removal efficiency to decrease as the initial concentration of the substance increases. On the other hand, time exhibits a positive but weak correlation with the removal percentage (r = 0.14), suggesting only a minor influence on removal efficiency. The temperature’s negative and moderate correlation (r = −0.28) with removal percentage may reflect a thermally dependent process where increased temperatures could potentially impede the removal efficiency. In the case of pH, there exists a moderately negative correlation with removal percentage (r = −0.40), hinting at a higher pH potentially reducing the efficacy of removal. Dosis, however, shows a negligible negative correlation with removal percentage (r = −0.06), indicating a very slight decrease in removal efficiency with increased dosis, though this relationship is so weak that it might not be significant. This correlation matrix not only highlights the strength and direction of relationships between multiple variables but also underscores the intricate dynamics within the removal process. The negative correlations observed with initial concentration and pH notably suggest that these variables are crucial for understanding the process in depth. While correlations provide insights into potential patterns within the dataset, they do not confirm causation and should prompt further investigative analysis, such as regression modeling, to unravel the complex interactions between these factors and optimize the conditions for maximum removal efficiency.

For the optimization [67] of removal percentage, predictive models of removal percentage, using a variety of machine learning algorithms, were evaluated for their effectiveness. The Gradient Boosting algorithm outshined its counterparts, exhibiting superior performance in terms of accuracy and computational efficiency. Henceforth, this discussion delineates the development process of a Gradient Boosting regression model, which is presented as the most efficacious algorithm for establishing a regression model between the input variables—’Initial concentration’, ‘Time’, ‘Temperature’, ‘pH’, and ‘Dosis’—and the target variable, ‘Removal percentage’. The Gradient Boosting Regressor was constructed using Python’s renowned Scikit-learn library, a tool of choice for its comprehensive suite of machine learning algorithms. The model was tailored with hyperparameters set to 100 estimators, ensuring a robust learning process without overfitting. The data division was configured with a traditional 80–20 split between the training and testing datasets, facilitated by Scikit-learn’s *train_test_split* function. Random state control was employed to ensure reproducibility of the results. This computational experiment was executed within a Python programming environment on Google Colab, renowned for its extensive support for data analysis and machine learning. The meticulous selection and tuning of hyperparameters were pivotal in harnessing the full potential of the Gradient Boosting method. The resultant model not only encapsulates the intricate relationships within the data but also stands as a testament to the meticulous computational methodology, paving the way for future endeavors in process optimization.

**Figure 13:**
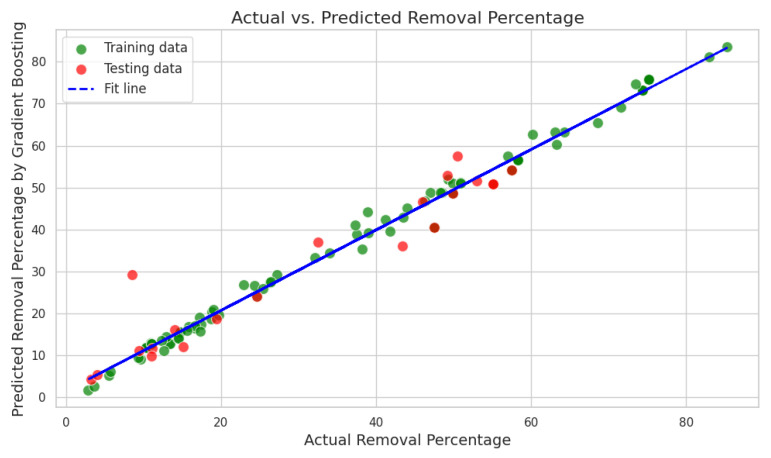
Regression fit model between the actual removal percentage and the predicted removal percentage obtained through the application of Gradient Boosting method.

Figure 13 shows a scatter plot that illustrates the relationship between the actual and predicted removal percentages derived from a Gradient Boosting regression model. The green dots represent predictions on the training dataset, while the red dots denote predictions on the testing dataset. A dashed blue line, described as the “fit line”, suggests a linear relationship indicative of the model’s predictions compared to the true values. Generally, the proximity of the dots to the fit line reflects the accuracy of the model: the closer the dots are to the line, the more accurate the predictions. In this case, both green and red dots are close to the fit line, indicating good model performance on both the training and testing sets. The continuation of the dots along the fit line without deviating significantly in either direction suggests that the model does not have a systemic bias toward over- or under-prediction. The blue fit line likely represents the ideal scenario where the predicted values exactly match the actual values, often referred to as the line of perfect fit. If the model’s predictions were perfect, all points would lie on this line, with predicted values equaling actual values. The training and testing data are clustered around a trend line defined by the equation *y* = 0.9583*x* + 1.5813. This equation embodies the model’s predictive behavior, where y represents the predicted removal percentage and x represents the actual removal percentage. The slope of the trend line, 0.9583, indicates that for every unit increase in the actual removal percentage, the predicted removal percentage increases by approximately 0.9583 units. This value, being close to 1, signifies that the model predictions are nearly proportional to the actual values, highlighting the model’s accuracy. The y-intercept of 1.5813 suggests that when the actual removal percentage is zero, the model predicts a baseline removal percentage of 1.5813, which might be interpreted as the model’s expected performance when no contributing factors are present. Overall, the proximity of the data points to the trend line, along with the slope nearing unity, reinforces the model’s precision and suggests minimal bias in prediction. However, the slight deviation from a perfect 1:1 slope implies that the model may slightly underestimate the removal percentage since the slope is less than 1. The y-intercept also suggests a small systematic offset in the model’s predictions. This detailed interpretation of the trend line equation allows for a nuanced understanding of the model’s characteristics and provides an avenue for further refining its predictions for use in optimization processes.

The Gradient Boosting (GB) model’s performance metrics for both training and testing phases offer a comprehensive evaluation of its predictive accuracy, as displayed in Table 11. The mean squared error (MSE) for the training set stands at approximately 4.05, suggesting a satisfactory model fit, whereas a notably higher MSE of around 33.17 for the testing set indicates potential overfitting, with the model not generalizing as well to unseen data. The sum of squares error (SSE) corroborates this, with values of 340.39 and 696.54 for the training and testing sets, respectively, indicating a greater cumulative deviation in the testing phase. The mean absolute percentage error (MAPE) values, 5.95% for training and 23.09% for testing, further reflect the model’s accuracy deterioration from training to testing. Root mean squared error (RMSE) follows the same pattern, with a lower training error of 2.01, contrasted with a higher testing error of 5.76, mirroring the MSE’s suggestion of overfitting. Similarly, the mean absolute error (MAE) is relatively low for training data at 1.49 but escalates to 3.70 for the testing data, highlighting larger average prediction errors when the model encounters new data. The mean percentage error (MPE) indicates an underprediction bias in both datasets, with more pronounced underestimation in the testing data at −14.27%, compared to −0.92% for training data. The coefficient of determination (COD), or R², which assesses the variance in the dependent variable explained by the independent variables, presents high values of 0.992 for training and 0.914 for testing. Despite the reduction in the testing phase, these values imply the model’s strong explanatory power. Overall, while the GB model exhibits robust predictive capabilities for the training data, evidenced by high R² and low error metrics, the escalation of error measures in the testing data suggests an imperative for model refinement to enhance its generalization and mitigate overfitting, ensuring more reliable predictions across new datasets.

**Figure 14:**
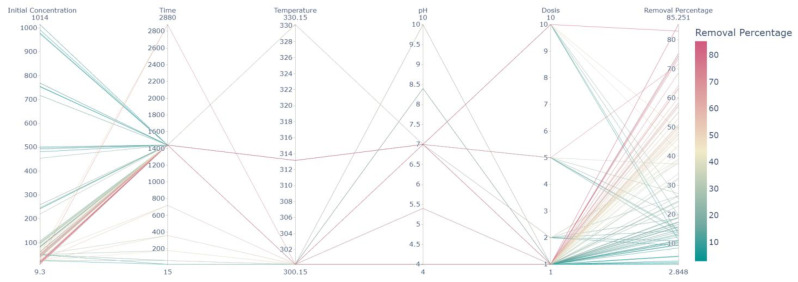
Parallel coordinate plot showing the multi-dimensional visualization of the data variables.

Figure 14 presents a parallel coordinates plot, a multidimensional visualization that allows for the inspection of individual data points across several quantitative variables simultaneously. The axes represent the variables under consideration, namely Initial Concentration, Time, Temperature, pH, Dosis, and Removal Percentage. Each line in the plot corresponds to a single observation from the dataset, with the position on each axis reflecting its value for the respective variable. Color gradation from turquoise to deep red indicates the spectrum of Removal Percentage values, serving as the classification criterion in this context. Observations with higher removal efficiencies are denoted in warmer hues, which suggests a tendency towards lower Initial Concentrations and possibly lower Dosis levels. Notably, there is no discernible trend relating Temperature or Time to removal efficiency, as lines are largely intermixed along these axes. Meanwhile, pH values predominantly cluster around neutral, with only a few observations indicating more extreme acidic or basic conditions. This visualization technique reveals complex relationships within the dataset that may not be apparent through traditional scatter plots or correlation matrices. It can be instrumental in identifying patterns, outliers, and potential areas of interest for further statistical analysis or experimental investigation. For instance, one could hypothesize from the visualization that for this particular removal process, optimal efficiency may be more sensitive to variations in initial concentrations and less affected by temperature or dosis variations within the examined range. Such insights are valuable for optimizing the parameters of the removal process to achieve higher efficiencies.

The problem is then solved for the optimization problem [68,69]. For the maximization of the removal percentage, the developed code implements a process known as Bayesian optimization using the scikit-optimize library to fine-tune the hyperparameters of a Gradient Boosting Regressor. The aim is to find the set of hyperparameters that maximizes the removal percentage predicted by the model.

Hyperparameters: These are the adjustable parameters that control the model training process. For Gradient Boosting, typical hyperparameters can include the number of trees (n_estimators), the learning rate, the depth of each tree, etc. The specific hyperparameters being optimized in this snippet are not directly shown, but we assume that they are encapsulated within the GradientBoostingRegressor with n_estimators = 100 and random_state = 42. The n_estimators parameter determines the number of sequential trees to be modeled, and the random_state ensures reproducibility of results.

Search Space: The search space is defined by a set of ranges for the input variables the model will use to make predictions. This space is represented as a list of Real or Integer objects, which are types provided by scikit-optimize to specify continuous and discrete parameters, respectively.

- ‘Initial concentration’ varies between 10 and 1000 (continuous).
- ‘Time’ varies between 15 and 2880 (discrete).
- ‘Temperature’ varies between 300.15 and 330.15 (continuous).
- ‘pH’ varies between 4 and 10 (continuous).
- ‘Dosis’ varies between 1 and 10 (continuous).

These parameters are part of the input feature set for the Gradient Boosting model, and the ranges provide boundaries for the Bayesian optimization algorithm to search within.

Optimization Algorithm: The following specific parameters are used for the Bayesian optimization algorithm.

- gp_minimize is the function from scikit-optimize that performs the Bayesian optimization. It utilizes Gaussian Processes to model the probability distribution of the objective function and makes educated guesses where the function might achieve optimal values.
- The objective function is defined to return the negative predicted removal percentage by the model given a set of parameters. The negative sign is used because gp_minimize by default searches for the minimum value of the function, but since we want to maximize the removal percentage, we need to minimize its negative.
- n_calls = 50 specifies the number of evaluations of the objective function, or how many times the algorithm will try different sets of parameters.
- random_state = 42 ensures that the results are reproducible; the algorithm will start from the same random seed.

**Figure 15:**
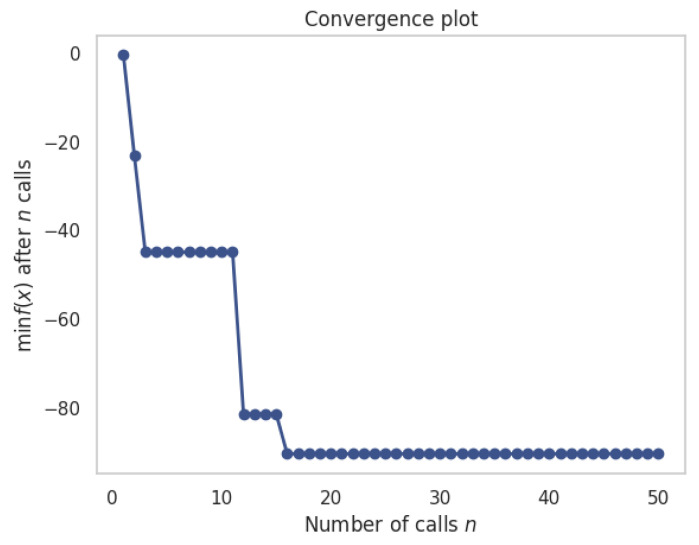
Convergence plot for the optimization process using Bayesian optimization algorithm.

The convergence plot, which is shown in Figure 15, visualizes the optimization algorithm’s progress over successive iterations, depicting the minimum objective function value attained after each function call. In the context of Bayesian optimization, each function call equates to an evaluation of the objective function with a particular set of hyperparameters. The y-axis, representing the minimum of the objective function, shows an initially steep decline, indicating a rapid improvement in the model’s performance with the first few evaluations. As the optimization proceeds, the curve flattens out, suggesting that subsequent iterations provide incremental improvements and eventually reach a plateau. This behavior is characteristic of optimization algorithms converging towards an optimal set of parameters. Around the 20th call, the curve levels significantly, implying that the algorithm has likely identified a region close to the optimum. Subsequent iterations refine the solution, but the objective function’s value changes only marginally, signifying that further searches yield little benefit, and the algorithm is approaching the maximum predicted removal percentage. This plateau indicates the optimal solution space within the predefined hyperparameter boundaries has been thoroughly explored, and additional evaluations do not substantially enhance the predictive capacity of the model. The figure encapsulates the efficiency and effectiveness of the Bayesian optimization process, demonstrating a pronounced convergence within 50 evaluations, which is critical in the context of resource-intensive computational tasks typical in scientific research.

Table 12 presents the set of input variables that yield the optimal removal percentage as determined by the optimization algorithm. The table outlines that an initial concentration of 10.0 units, a lengthy process time of 2880 units, a mid-range temperature of 313.15 units, a relatively acidic pH of 4.0, and the lowest dosis level of 1.0 unit culminate in a removal percentage of approximately 90.4733%. This optimal scenario likely reflects a specific operational sweet spot for the system under study, balancing the chemical and physical inputs to maximize efficiency. The removal percentage achieved underscores the model’s capability to identify conditions under which the process efficiency is near its peak within the constraints and ranges specified in the study.

## 3. Materials and Methods

### 3.1. Adsorbate

**Figure 16:**
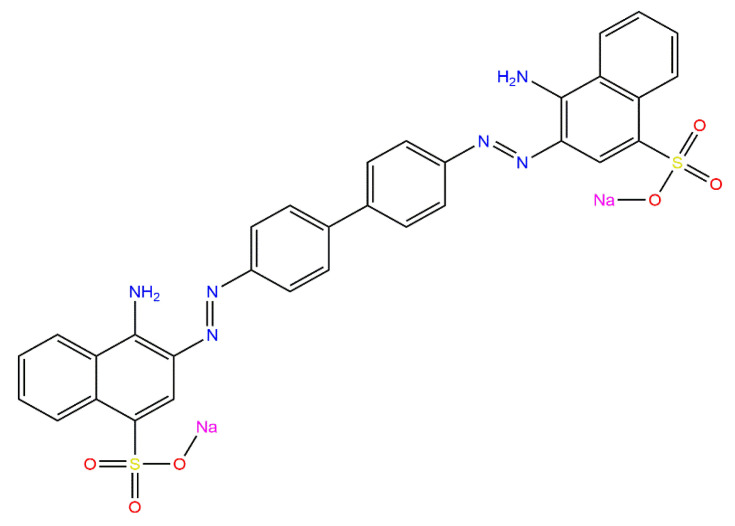
Chemical structure of Congo red (CR) (ChemDraw professional software).

Congo red dye (C.I. 22120, CAS number: 573-58-0, chemical formula: C_32_H_22_N_6_Na_2_O_6_S_2_) was reagent grade (99.92%) and procured from Fagalab. Figure 16 shows the CR molecule (drawn with ChemDraw professional software 17 version 17.0.0.206), and Table 13 presents the physical properties of CR dye.

### 3.2. Biochar Preparation

The bark of *Haematoxylum campechianum* was collected from the Faculty of Engineering, Autonomous University of Carmen in Campeche, Mexico. First, a pretreatment was carried out. The bark was chopped, milled, and sieved between 0.2 and 0.5 mm. Then, it was washed with abundant distilled water at 50 °C to eliminate any residue from the surface and oven-dried at 70 °C for 12 h.

For the chemical activation, 250 mL of H_3_PO_4_ was mixed with 50 g of *Haematoxylum campechianum* (pretreated, previously) for 3 h at 50 °C, and then, the mixture was filtered and the solids dried at 70 °C for 12 h. For the thermal treatment, 50 g of phosphoric acid-treated *Haematoxylum campechianum* was introduced into a muffle at 500 °C for 60 min, at 10 °C/min. Then, when the sample was cold, in order to remove H_3_PO_4_ residue, the activated biochar was washed with a 5% NaHCO_3_ solution and then with abundant distilled water until the filtrate reached a pH value between 6 and 7. Finally, activated biochar from *Haematoxylum campechianum* (*ABHC*) was dried at 110 °C for 12 h and then stored in a closed glass bottle and placed in a desiccator.

### 3.3. Characterization Techniques

Scanning electron microscope (SEM) coupled with EDS system (HITACHI S-3400N), Brunauer Emmett Teller method (BET) (BELSORP MAX), X-ray diffractometer (XRD) (APD 2000 PRO), and Fourier-transform infrared (FTIR) spectrophotometer (Nicolet Nexus 670) techniques were used to characterize the surface morphology, shape, elemental composition, mean pore diameter, surface area, crystalline structure, and chemical composition of the activated biochar from *Haematoxylum campechianum* (*ABHC*). Additionally, the pH point of zero charge pH_pzc_ was determined using 0.01 M NaCl as the background electrolyte solution; the pH of the working solutions was fixed between 1 and 12 (2, 4, 5, 6, 8, 10, and 12) using 0.1 M NaOH and/or HCl. First, the initial pH of each solution was measured with a Hanna instruments (model HI2020-01) pH meter. Subsequently, solutions were put in contact with the adsorbent at 120 rpm at 300.15 K for 24 h. Then, the solutions were decanted, and the final pH values were measured. The point of intersection between the curve was obtained by plotting pH_final_ vs. pH_initial_, and the diagonal curve corresponds to the pH_pzc_ of the adsorbent [70].

### 3.4. Sorption

Sorption of Congo red is affected by several parameters, including the pH of the aqueous medium, temperature, adsorbent dose, adsorbate concentration, and contact time.

Different pH values (4.0, 5.4, 7.0, 8.4, and 10.2) of CR solutions were adjusted by adding 0.1 M HCl and 0.1 M NaOH, using a pH sensor (model HI2020-01, Hanna instruments) at different initial concentrations (10, 20, 40, 50, 60, 80, and 100 mg/L) and put in contact with a dose of activated biochar =1 g/L in a rotating incubator at 150 rpm for 24 h at 300.15K.

The effect of the temperature was evaluated by putting in contact with different initial concentration CR solutions (20, 50, 100, 250, 500, 750, and 1000 mg/L) with a dose of *ABHC* =1g/L, pH solution = 7.0 at three different temperatures: 300.15K, 313.15K, and 330.15K for 6 h.

In order to evaluate the effect of the dose of adsorbent material on the dye adsorption, four doses of *ABHC* (1, 2, 5, and 10 g/L) were put in contact for 24 h with different initial concentration CR solutions (20, 50, 100, 250, 500, 750, and 1000 mg/L) at pH solution = 7.0 and temperature = 300.15 K.

After reaching equilibrium, all samples were centrifugated for 5 min at 3500 rpm and then analyzed by a UV-vis spectrophotometer (model EVOLUTION 220, Thermo Scientific) using calibration curves (with an interval from 2 to 20 mg/L) and R^2^ > 0.995 at the wavelength of 497 nm.

The adsorption capacity (q_e_) was calculated using the following Equation:

(2) $$q_{e}=\frac{\left(C_{i}-C_{e}\right)\cdot V}{m}$$

where *C_i_* (mg/L) is the initial concentration, *C_e_* (mg/L) is the equilibrium concentration, *q_e_* (mg/g) is the adsorption capacity, *V* (L) is the volume, and *m* (g) is the mass of the adsorbent.

Experimental equilibrium data were fitted with the following nonlinear isotherms models: Langmuir (Equation (3)), Freundlich (Equation (4)), and Redlich–Peterson (Equation (5)), using the solver add-in from Microsoft’s spreadsheet tool of Microsoft Excel for Office 365 version 2403.

(3) $$q_{e}=\frac{Q_{max}K_{L}C_{e}}{1+K_{L}C_{e}}$$

where *K_L_* (L/mg) is the Langmuir equilibrium constant and *Q_max_* (mg/g) is the maximum adsorption capacity.

(4) $$q_{e}=K_{F}C_{e}^{1/n}$$

where *n* (dimensionless) and *K_F_* (mg^1−1/n^·L^1/n^·g^−1^) are the exponent and the Freundlich parameter, respectively.

(5) $$q_{e}=\frac{K_{RP}C_{e}}{1+\alpha_{RP}C_{e}^{\beta }}$$

where *K_RP_* (L/g) and *α_RP_* (L/mg) are the constant of the Redlich–Peterson and β is a dimensionless parameter between 0 and 1.

Kinetic studies were conducted from two different initial concentrations: 25 mg/L and 50 mg/L at pH = 7.0, an adsorbent dose = 1 g/L, temperature = 300.15 K, and the following contact times: 15, 60, 180, 360, 720, 1440, and 2880 min. The obtained experimental data were fitted with the pseudo-first-order (PFO), pseudo-second-order (PSO), and Elovich kinetic nonlinear models (Equation (6), Equation (7), and Equation (8), respectively) using the solver add-in from Microsoft’s spreadsheet tool of Microsoft Excel.

(6) $$q_{t}=q_{e,cal}\left(1-exp(-k_{1}t\right))$$

(7) $$q_{t}=\frac{k_{2}q_{e,cal}^{2}t}{1+k_{2}q_{e,cal}t}$$

where *q_e_,_cal_* is the theoretical adsorption capacity (mg/g) and *k*_1_ (1/min) and *k*_2_ [g/(mg·min)] are the pseudo-first and pseudo-second-order rate constants, respectively.

(8) $$q_{t}=\frac{1}{\beta }ln(\alpha \beta t)$$

where *β* is the constant related to the extent of surface coverage (g/mg) and α is the theoretical adsorption capacity [mg/(g·min)].

In addition to the regression coefficient R^2^, the following error functions were calculated with the aim of confirming accurate measurement results and a good fit with the proposed models: absolute error (EABS), sum squared error (SSE), the average percentage error (ARE), nonlinear chi-square test (χ^2^), residual root mean square error (RMSE), normalized standard deviation (Δ*q* (%)) (Equation (9), Equation (10), Equation (11), Equation (12), Equation (13) and Equation (14), respectively).

(9) $$SSE=\sum_{i=1}^{N}{{(q}_{e,exp}-q_{e,calc})}^{2}$$

(10) $$EABS=\frac{1}{n}\sum_{i=1}^{N}\left|{(q}_{e,exp}-q_{e,calc}\right|$$

(11) $$ARE\left(\%\right)=\frac{100}{N}\sum_{i=1}^{N}\left|\frac{q_{e,exp}-q_{e,calc}}{q_{e,exp}}\right|$$

(12) $$\chi^{2}=\sum_{i=1}^{N}\frac{{{(q}_{e,exp}-q_{e,calc})}^{2}}{q_{e,calc}}$$

(13) $$RMSE=\sqrt{\frac{1}{N-2}\sum_{i=1}^{N}{{(q}_{e,exp}-q_{e,calc})}^{2}}$$

(14) $$\Delta q(\%)=100\sqrt{\frac{1}{N-1}\sum_{i=1}^{N}({\frac{q_{e,exp}-q_{e,calc}}{q_{e,exp}})}^{2}}$$

where *q_e,exp_* is the adsorption capacity obtained from the batch experiment (mg/g), *q_e,calc_* is the adsorption capacity obtained with a mathematical model corresponding (mg/g), and *N* is the corresponding number of observations in the experiment.

### 3.5. Desorption and Regeneration Study

In order to determine the desorption behavior and reusability of *ABHC*, 0.01 M and 0.2 M NaOH solutions were used as desorbing agents. A total of 50 mL of CR at the initial concentration of 100 mg/L was put in contact with 50 mg of *ABHC* for 24 h. Then, CR-loaded *ABHC* was regenerated using 50 mL of NaOH solution for CR desorption. Regeneration was carried out at 300.15 for 24 h. After a time, the sample was centrifuged to obtain the adsorbent. The residual concentration was measured by UV-vis spectrophotometric analysis. The regenerated *ABHC* was rinsed with distilled water and dried at 105 °C for 2 h before the next cycle of adsorption took place. Two cycles of consecutive adsorption–desorption studies were carried out using two different concentrations of NaOH (0.01 M and 0.2 M).

*Q_des_* (mg/g) is the quantity of dye desorbed that was calculated using Equation (15) [71].

(15) $$Q_{des}=C_{des}\cdot \frac{V}{m}$$

where *C_des_* (mg/L) is the concentration of dye left after desorption, *V* (L) is the volume of the dye solution, and *m* (g) is the *ABHC* mass.

*Q_ads_* (mg/g) is the quantity of dye adsorbed after sorption that was calculated using Equation (2).

## 4. Conclusions

According to the characterization, isotherm, and kinetic results, the interactions involved in the CR removal process onto biochar prepared from *Haematoxylum campechianum* waste (*ABHC*) were electrostatic attraction, hydrophobic attraction, hydrogen bonding, and n–π and π–π interactions. The results of the evaluated effects (pH, temperature, and dose) give a maximum adsorption capacity (Q_max_) equal to 114.8 mg/g at pH = 5.4, 300.15K, and a dose of *ABHC* of 1 g/L.

Advanced machine learning techniques including Gradient Boosting Model combined with an optimization algorithm were applied to optimize a chemical removal process. The resulting model predicted a removal percentage of approximately 90.47% under optimal conditions: an initial concentration of 10 units, time of 2880 units, temperature at 313.15 units, pH of 4.0, and dosis of 1.0.

Finally, this study underscores the vital role of computational thinking in addressing environmental challenges through the implementation of computational packages such as data science, machine learning, and optimization. This work not only contributes to the field of environmental science but also provides valuable insights for integrating computational thinking skills into educational curricula. We can foster innovation and sustainability, empowering individuals to tackle real-world environmental problems with confidence and efficacy.

## Figures and Tables

**Table 1:** FT-IR bands before and after the adsorption process.

Functional Group	Wavenumber (cm^−1^)	
	*ABHC*	*ABHC*-CR
O-H and N-H	3358	3368
C=O	1705	1705
C=C	1593	1600
C-C (in aromatic ring)	-	1421
C-O	-	1360
C-O	1224	1196
S=O	-	1045
N-H	779	769

**Table 2:** Kinetic parameters of CR adsorption onto (*ABHC*) at different C_i_ values.

C_i_ (mg/L)	Pseudo-First-Order				Pseudo-Second-Order			Elovich		
	q_e,exp_ (mg/g)	q_e_ (mg/g)	k_1_/10^−2^ (1/min)	R^2^	q_e_ (mg/g)	k_2_/10^−3^ [g/(mg·min)]	R^2^	α [mg/(g·min)]	β (g/mg)	R^2^
25	15.21	13.87	1.187	0.957	15.09	1.056	0.988	0.8100	0.4183	0.928
50	23.74	21.95	0.5693	0.877	23.81	0.3724	0.939	0.7880	0.2590	0.968

**Table 3:** Error functions data of the kinetic models.

C_i_ (mg/L)	Model	Error Functions					
		ARE	SSE	∆q (%)	χ^2^	EABS	RMSE
25	PFO	7.593	5.918	8.996	0.512	0.819	1.088
	PSO	7.669	1.599	15.085	0.373	0.389	0.565
	Elovich	20.518	9.891	41.892	2.617	1.033	1.406
50	PFO	19.160	37.763	32.024	4.476	1.974	2.748
	PSO	12.081	18.762	23.587	2.331	1.198	1.937
	Elovich	7.818	10.001	10.337	0.693	1.040	1.414

**Table 4:** Parameters of the Langmuir, Freundlich, and Redlich–Peterson nonlinear models for the adsorption of CR onto activated biochar from *Haematoxylum campechianum* (*ABHC*) at different pH values.

Isotherm Models	Parameters	pH				
		4	5.4	7	8.4	10.2
Langmuir	Q_max_ (mg/g)	53.93	114.8	68.33	30.83	10.51
	K_L_/10^−2^ (L/mg)	7.252	1.108	3.679	0.832	9.695
	R^2^	0.982	0.994	0.972	0.988	0.962
	n	2.479	1.335	1.830	1.362	3.4315
Freundlich	K_F_ (mg/g)(L/mg)^1/n^	8.945	2.189	5.393	0.498	2.684
	R^2^	0.940	0.990	0.992	0.983	0.941
Redlich–Peterson	α_RP_ (L/mg)	0.085	0.001	1.726	0.015	0.214
	K_RP_ (L/g)	4.088	1.141	12.761	0.270	1.395
	β	0.973	1.453	0.516	0.887	0.895
	R^2^	0.982	0.995	0.992	0.988	0.969

**Table 5:** Error functions data of the pH effect.

pH	Model	Error Functions					
		ARE	SSE	∆q (%)	χ^2^	EABS	RMSE
4.0	Langmuir	4.440	11.51	5.922	0.394	1.161	1.696
	Freundlich	11.49	37.99	19.43	2.439	2.214	3.082
	Redlich–Peterson	4.731	11.41	6.443	0.421	1.186	1.689
5.4	Langmuir	6.050	6.119	9.495	0.495	0.816	1.106
	Freundlich	5.246	10.64	7.910	0.557	1.092	1.459
	Redlich–Peterson	5.997	5.692	10.97	0.565	0.710	1.067
7.0	Langmuir	10.32	32.09	15.52	1.933	1.811	2.533
	Freundlich	6.060	8.534	9.491	0.602	0.947	1.306
	Redlich–Peterson	5.317	8.615	8.012	0.520	0.899	1.313
8.4	Langmuir	5.612	1.246	7.802	0.153	0.354	0.499
	Freundlich	9.324	1.672	16.82	0.382	0.407	0.578
	Redlich–Peterson	5.818	1.237	8.731	0.166	0.350	0.497
10.2	Langmuir	4.587	1.049	5.899	0.143	0.325	0.458
	Freundlich	6.917	1.617	11.29	0.334	0.386	0.569
	Redlich–Peterson	4.611	0.836	6.531	0.139	0.287	0.409

**Table 6:** Parameters of the Langmuir, Freundlich, and Redlich–Peterson nonlinear models for the adsorption of CR onto different doses of activated biochar from *Haematoxylum campechianum* (*ABHC*).

Isotherm Models	Parameters	Dose (g/L)			
		1	2	5	10
Langmuir	Q_max_ (mg/g)	92.86	59.53	47.62	12.22
	K_L_/10^−2^ (L/mg)	1.887	0.369	0.125	0.685
	R^2^	0.940	0.988	0.970	0.966
	n	3.079	2.051	1.579	2.551
Freundlich	K_F_ (mg/g)(L/mg)^1/n^	10.85	1.747	0.346	0.787
	R^2^	0.976	0.993	0.985	0.982
Redlich–Peterson	α_RP_ (L/mg)	0.401	0.091	252.8	3.338
	K_RP_ (L/g)	6.788	0.506	87.64	2.811
	β	0.742	0.669	0.367	0.618
	R^2^	0.984	0.997	0.985	0.982

**Table 7:** Error functions data of the dose effect.

Dose (g/L)	Model	Error Functions					
		ARE	SSE	∆q (%)	χ^2^	EABS	RMSE
1	Langmuir	14.52	552.4	22.17	10.83	5.004	7.835
	Freundlich	16.67	224.9	32.14	10.60	3.950	4.999
	Redlich–Peterson	7.262	151.7	10.66	2.813	2.886	4.105
2	Langmuir	12.22	19.55	20.21	1.881	1.355	1.977
	Freundlich	9.945	12.46	19.84	1.215	1.083	1.579
	Redlich–Peterson	4.830	4.912	6.941	0.332	0.698	0.991
5	Langmuir	26.89	14.41	43.57	3.437	1.215	1.698
	Freundlich	13.67	7.352	26.16	1.264	0.777	1.213
	Redlich–Peterson	13.68	7.356	26.18	1.266	0.778	1.213
10	Langmuir	20.93	2.996	36.48	1.346	0.573	0.774
	Freundlich	9.020	1.549	13.55	0.315	0.363	0.557
	Redlich–Peterson	9.977	1.543	14.89	0.342	0.375	0.556

**Table 8:** Parameters of the Langmuir, Freundlich, and Redlich–Peterson nonlinear models for the adsorption of CR onto activated biochar from *Haematoxylum campechianum* (*ABHC*) at different temperatures.

Isotherm Models	Parameters	Temperature (K)		
		300.15	313.15	330.15
Langmuir	Q_max_ (mg/g)	92.86	27.44	29.20
	K_L_/10^−2^ (L/mg)	1.887	26.78	1.917
	R^2^	0.940	0.709	0.980
	n	3.079	7.411	3.878
Freundlich	K_F_ (mg/g)(L/mg)^1/n^	10.85	12.38	5.106
	R^2^	0.976	0.999	0.951
Redlich–Peterson	α_RP_ (L/mg)	0.401	19.66	0.057
	K_RP_ (L/g)	6.788	247.2	0.852
	β	0.742	0.867	0.899
	R^2^	0.984	0.999	0.988

**Table 9:** Error functions data of temperature effect.

Temperature (K)	Model	Error Functions					
		ARE	SSE	∆q (%)	χ^2^	EABS	RMSE
300.15	Langmuir	14.52	552.4	22.17	10.83	5.004	7.835
	Freundlich	16.67	224.9	32.14	10.60	3.950	4.999
	Redlich–Peterson	7.262	151.7	10.66	2.813	2.886	4.105
313.15	Langmuir	13.64	71.57	16.61	3.316	2.978	3.783
	Freundlich	0.813	0.321	1.211	0.017	0.162	0.254
	Redlich–Peterson	0.673	0.289	1.054	0.014	0.145	0.240
330.15	Langmuir	6.921	8.183	14.16	0.958	0.746	1.279
	Freundlich	9.950	20.13	13.09	1.245	1.580	2.006
	Redlich–Peterson	5.720	4.807	8.774	0.433	0.752	0.980

**Table 10:** Comparison of the maximum adsorption capacity of Congo red onto different activated carbons.

Substrate	Q_max_ (mg/g)	T (°C)	pH	C_i_ (mg/L)	References
Coffee waste	90.90	25	3.0	20–120	[35]
Kenaf fiber (*Hibiscus cannabinus*)	14.20	27	7	5–25	[33]
Guava leaves	47.62	30	3	10–50	[52]
Rubber (*Hevea brasiliensis*)	55.87	30	2	100–500	[36]
Aloe vera leaves	91.00	25	2	100	[53]
*Cornulaca monacantha*	78.19	55	2.0	20–160	[54]
Peanut shell	153.4	-	-	20–200	[50]
Casuarinas waste	232.0	25	-	5–1000	[51]
*Delonix regia*	17.12	30	-	200–1200	[55]
Corn cobs	41.67	50	3	10–50	[56]
*Haematoxylum campechianum*	114.8	27	5	10–100	This study

**Table 11:** Performance metrices of Gradient Boosting method during the training and testing phase.

Type of Data	MSE	SSE	MAPE	RMSE	MPE	COD
Training data	4.052313	340.3943	5.947051	2.013036	−0.92448	0.992258
Testing data	33.1685	696.5386	23.08819	5.75921	−14.2749	0.91353

**Table 12:** Optimal removal percentage and the input variables.

Initial Concentration (mg/L)	Time (min)	Temperature (K)	pH	Removal Percentage
10.0	2880	313.15	4.0	90.4733

**Table 13:** Physical properties of Congo red (CR).

Parameter	Value
Molecular weight	696.66 g/mol
Density	0.995 g/mL at 25 °C
Solubility	H_2_O: 25 g/L
Water solubility pK_a_	Soluble 4.5 [44]
pH	6.7 (10 g/L, H_2_O and at 20 °C)
Color and pH range	3 (blue)–5.2 (red)
λ_max_	567 nm at pH 2.18–3.16 and 497 nm at pH ≥ 3.86

## Data Availability

The data presented in this study are available on request from the corresponding author.
